# The effect of a performance-based financing program on HIV and maternal/child health services in Mozambique—an impact evaluation

**DOI:** 10.1093/heapol/czx106

**Published:** 2017-10-23

**Authors:** Yogesh Rajkotia, Omer Zang, Pierre Nguimkeu, Jessica Gergen, Iva Djurovic, Paula Vaz, Franscisco Mbofana, Kebba Jobarteh

**Affiliations:** 1 ThinkWell, Av. Cahora Bassa, Nr 122 2, Andar (2nd Floor) Bairro da Somerschield Maputo, Maputo, Mozambique; 2 Fundação Ariel Glaser Contra o SIDA Pediátrico, Maputo, Mozambique; 3 Direcção Nacional de Saude Publica, Ministerio da Saude, Maputo, Mozambique; 4 Centers for Disease Control and Prevention, Maputo, Mozambique

**Keywords:** Performance-based financing, impact evaluation, maternal and child health, Mozambique

## Abstract

Performance-based financing (PBF) is a mechanism by which health providers are paid on the basis of outputs or results delivered. A PBF program was implemented on the provision of HIV, prevention of mother-to child HIV transmission (PMTCT), and maternal/child health (MCH) services in two provinces of Mozambique. A retrospective case–control study design was used in which PBF provinces were matched with control provinces to evaluate the impact of PBF on 18 indicators. Due to regional heterogeneity, we evaluated the intervention sites (North and South) separately. Beginning January 2011, 11 quarters (33 months or 2.75 years) of data from 134 facilities after matching (84 in the North and 50 in the South) were used. Our econometric framework employed a multi-period, multi-group difference-in-differences model on data that was matched using propensity scoring. The regression design employed a generalized linear mixed model with both fixed and random effects, fitted using the seemingly unrelated regression technique. PBF resulted in positive impacts on MCH, PMTCT and paediatric HIV program outcomes. The majority of the 18 indicators responded to PBF (77% in the North and 66% in the South), with at least half of the indicators demonstrating a statistically significant increase in average output of more than 50% relative to baseline. Excluding pregnant women, the majority of adult HIV treatment indicators did not respond to PBF. On average, it took 18 months (six quarters) of implementation for PBF to take effect, and impact was generally sustained thereafter. Indicators were not sensitive to price, but were inversely correlated to the level of effort associated with marginal output. No negative impacts on incentivized indicators nor spill-over effects on non-incentivized indicators were observed. The PBF program in Mozambique has produced large, sustained increases in the provision of PMTCT, paediatric HIV and MCH services. Our results demonstrate that PBF is an effective strategy for driving down the HIV epidemic and advancing MCH care service delivery as compared with input financing alone.

Key Messages

- This study demonstrates that, in Mozambique, PBF is a superior strategy than standard input-based financing alone for improving MCH, PMTCT and paediatric HIV outcomes.
- Our findings show that PBF not only delivers large increases in health service outputs for many indicators, but that the effect of PBF is generally sustained over time. This study also takes a more robust view of whether PBF is effective by creating a responsiveness index that goes beyond simple statistical significance and accounts for the magnitude and duration of effect.
- Policymakers in Mozambique should consider scaling-up this program nationally, and potentially expanding to include other public health priorities including malaria.
- Using an exposure-time model, our study shows that the positive effects of PBF can be observed after six quarters of implementation, and are generally sustained thereafter.
- This study also takes a more robust view of whether PBF is effective by creating a responsiveness index that accounts for the magnitude and duration of statistically significant impact, an approach we recommend future impact evaluations build from.
- Policymakers as well as international funding organizations operating in Mozambique should consider scaling-up PBF programs nationally, and potentially expanding to include other public health priorities.
- Finally, this study applied the same model of PBF in two separate geographies of Mozambique, thus offering insights into the contextual factors that determine effectiveness. While other countries should take note of Mozambique’s results, they should also recognize that context is essential—results on the same indicators varied across regions—so policymakers must attend to their own varied contexts when designing PBF programs.

## Introduction

### Background

Mozambique has made remarkable progress on maternal and child health (MCH) in the past decade. Between 1997 and 2011, it has halved infant mortality from 147 to 71 deaths per 1000 live births and reduced maternal mortality from 692 to 408 per 100 000 live births (Ministry of Health (MISAU) 2011). While these gains are impressive, the devastating impact of the generalized HIV epidemic has stagnated progress in the health sector. In 2014, with an adult HIV prevalence rate of 10.6% and an estimated 1.5 million people living with HIV, Mozambique had the fifth largest HIV epidemic in Sub-Saharan Africa (Singh *et al.* 2014; UNAIDS 2014, 2015). Low motivation and poor retention of health workers, poor infrastructure and lack of essential medical supplies has contributed to the country’s inability to fully respond to its health challenges, including HIV care. A recent assessment of the health sector in Mozambique cited critical needs in performance improvement of service delivery (WHO 2013).

Mozambique’s health system has traditionally been financed through centralized input provision. Under an input financing framework, inputs such as equipment, drugs and provider salaries are provided on a regular basis to finance the delivery of care. The effectiveness of this arrangement has been criticized on the grounds that the link between funding and results is tenuous, as health care providers have limited financial incentive to maximize productivity (Soeters 2013). Proponents of performance-based financing (PBF) hold that financial incentives can increase outputs, quality and even coverage of health services through the use of financial incentives to motivate healthcare providers (Soeters 2013). At the same time, critics point to the risk of crowding out health workers’ intrinsic motivation with financial incentives, adverse impacts on non-rewarded services, and the long-term sustainability of such mechanisms (Lemière *et al.* 2012; Magrath and Nichter 2012). Globally, the evidence base on the effectiveness of PBF is limited and shows mixed findings (Martinez *et al.* 2012l Witter *et al.* 2012; Gorter *et al.* 2013; Renmans *et al.* 2016). A 2009 Cochrane review, which examined nine studies, found that no general conclusion could be made regarding the likely impact of PBF in low- and middle-income countries (Witter *et al.* 2012). The few rigorous studies that do exist largely focus on whether PBF ‘worked’ by examining whether there was a statistically significant change in the production of incentivized indicators from baseline values (Basinga *et al.* 2011; Bonfrer *et al.* 2014; Matsuoka *et al.* 2014; Skiles *et al.* 2015; Engineer *et al.* 2016). To our knowledge no rigorous impact evaluations have studied the lifecycle of PBF treatment effects over time, such as time needed to attain peak treatment effect and degradation of treatment effect over time. Similarly, the evidence base on the determinants of indicator responsiveness is limited. Moreover, few studies have examined the relationship between determinants of responsiveness and marginal output of incentivized indicators. By our own count, over 35 countries have implemented, are in the process of implementing or have planned to implement a PBF program to improve the performance of their health sectors (Bonfrer et al, 2014). Thus, rigorous evidence is essential to inform countries on how to best design and refine their PBF models to accelerate health sector performance.

In 2011, against this backdrop, a clinical NGO partner, funded by the United States Centres for Disease Control and Prevention (CDC), began implementing a PBF program in two provinces of Mozambique. As of 2014, the program has provided approximately $11 million in incentives and scaled up to cover most of Gaza and Nampula provinces. The program continues to be implemented but is not actively scaling-up (expansion geographically or population coverage).

The principal objective of this article is to evaluate whether PBF is a more effective alternative to input-based financing alone, with focus on HIV and MCH services. The secondary objective is to determine the temporal effects of PBF in order to better understand its lifecycle—how long it takes for PBF to take effect and how long this effect lasts. Third, this study examines the relationship between the level of effort for a particular indicator and its responsiveness to PBF. Finally, this study aims to determine whether there were any spill-over effects of PBF on non-incentivized services.

### PBF model

Starting in January 2011, health facilities in the provinces of Nampula (North) and Gaza (South) were enrolled into the PBF program in a phased manner over 18 months, with priority given to high-volume facilities. In order to enrol into the program, facilities needed to be certified by the Government of Mozambique (GRM) to provide both antiretroviral therapy (ART) and Prevention of Mother-to-Child Transmission of HIV (PMTCT) services. This PBF program is one of the only globally to target predominantly HIV services and the only one currently funded by CDC. The donor, program implementer (NGO), and provincial health directorates jointly selected 21 incentivized indicators, clustered in four groups by the type of service: PMTCT, Paediatric HIV, Adult HIV/Tuberculosis (TB) and MCH (Table 1). Payment is based on the unit price of a service multiplied by the quantity of that service produced. An equity weight was applied to favour facilities in rural and hard-to-reach areas.

**Table 1.** List of PBF and non-incentivized indicators and policy changes

Indicator description	Relevant policy changes during the study period
PMTCT	
Number of HIV-infected pregnant women who received antiretroviral (ARV) prophylaxis to reduce risk of mother-to-child transmission	National policy to increase the CD4 threshold for initiating ART from 250 to 350 was uniformly implemented in all four study provinces starting in January 2012 to June 2013. During the last quarter of the study period (June–September 2013), a national Option B+ policy for all pregnant women was passed and partially implemented in all four study provinces. [Option B+ is a WHO-approved policy for giving lifelong ART to all pregnant women] Secondly, the Ministry of Health (MOH) adopted the One Stop Model, promoting ART initiation for pregnant women in ANC by MCH nurse, as a national policy in January 2013. Some provinces started implementing this approach prior: Gaza in 2006, Nampula in 2011, Maputo in September 2012 and Cabo Delgado in December 2012, possibly introducing a bias in favour of controls
Number of HIV-infected pregnant women who initiated antiretroviral therapy (ART)	
Number of HIV-infected women who received an FP consultation and a modern contraceptive method	
Paediatric HIV	
Number of polymerase chain reaction (PCR) tests for HIV, administered to newborns 4–8 weeks of age born to an HIV-infected woman	
Number of HIV rapid tests administered to children 9–12 months of age born to an HIV-infected woman	
Number of HIV-infected children 0–23 months of age who initiated ART treatment for the first time	During the study period, the national policy stated that all HIV infected children under the age of 2 initiate ART treatment, regardless of clinical or immunologic eligibility
Number of HIV-infected children 2–14 years of age who initiated ART treatment for the first time	National policy to increase the CD4 threshold for initiating ART treatment from 250 to 350 was uniformly implemented in the four study provinces starting in January 2012
Number of HIV-infected children 0–14 years of age alive 12 months after initiating ART	
Adult HIV care and treatment	
Number of HIV-infected adults (excluding pregnant women) who initiated ART treatment for the first time	National policy to increase the CD4 threshold for initiating ART treatment from 250 to 350 was uniformly implemented in the four study provinces starting in January 2012
Number of adults co-infected with HIV and tuberculosis (TB) who initiated ART treatment for the first time	National policy to initiate ART for every patient co-infected with TB and HIV, regardless of clinical or immunologic eligibility, was uniformly implemented in the four study provinces starting in January 2012
Number of HIV-infected patients who initiated Isoniazid to prevent TB (all HIV infected patients are eligible)	
Number of HIV-infected adults (>15 years) alive 12 months after initiating ART	Community Adherence and Support Groups (GAAC) were piloted uniformly in all four study provinces starting in 2012 to improve ART treatment adherence
MCH	
Number of pregnant women who completed four ANC visits	
Number of pregnant women who delivered at the health facility	
Number of children who receive full vaccination for Bacillus Calmette–Guérin (BCG), tetanus, diphtheria, and pertussis (DPT), polio and measles in the first 9 months	
Number of women, excluding HIV-infected women, who received an FP consultation and a modern contraceptive method	
Number of post-natal consultations a women receives 3–28 days after birth	
Number of children with acute malnutrition who completed treatment and satisfied the defined clinical criteria for discharge	
Non-incentivized indicators	
Number of women who have received the three recommended doses of malaria prophylaxis during the second and third trimester of pregnancy	
Number of well child consultations in the first 4 years of age	
Excluded indicators	
Number of HIV-infected patients lost to follow up who come back for ART treatment	
Number of male partners (of all women) tested for HIV	
Number of HIV tests administered at the health facility	

*Sources*: MOH policy documents, Boletins da República

Health facilities report on PBF indicators monthly and submit aggregated reports, based on facility registers, on a quarterly basis. Data verification and payment cycles are scheduled for every quarter and conducted jointly by the program implementer and the provincial health department. Verification is comprehensive—every indicator at all facilities is verified. Standard operating procedures guide the verification process. First, reported outputs are cross-checked with source register books. Second, facility register data are cross-checked with patient files and pharmacy records. Strong financial penalties are in place to dissuade inaccurate reporting. A 10% variance between reported and verified reports for a particular indicator results in non-payment for that indicator. If a 10% discrepancy is found in a third or more of the indicators in a particular health facility, that health facility forfeits all PBF payments for that quarter.

PBF earnings are allocated to facility investment (40%) and salary top-ups (60%). Salary top-ups are distributed among health facility staff based on a pre-determined criteria including years of experience and level of education. According to internal program data, salary top-ups account for between 20 and 50% of an average health workers salary, and facility investments account for approximately 50% of total facility operating costs.

## Methods

### Study design

While the PBF model and its implementation were identical in the northern and southern provinces, there is a high degree of heterogeneity in sociodemographic factors, including HIV prevalence and health services uptake between the provinces themselves (Table 2). Therefore, we chose to treat each region as a separate experiment so as to allow for inferences on how the performance of PBF differs by context.

**Table 2.** Profile of study provinces

	Southern region		Northern region	
	Gaza (PBF intervention)	Maputo* (input financing)	Nampula (PBF intervention)	Cabo Delgado (input financing)
Provincial characteristics				
Provincial GDP per capita (USD)^e^	336	1,016	320	253
Population^b^	1,315,732	1,444,623	4,529,804	1,764,194
Gini coefficient^d^	0.33	0.21	0.42	0.49
% women entering secondary school^c^	18.5	37.2	10.4	7.3
Health professionals per 100 000 inhabitants^a^	103.6	168.4	77.3	75.8
Annual outpatient visits per capita^b^	1.47	1.47	0.95	1.22
Population per facility (levels 1 and 2)^c^	11,245	18,057	23,716	18,001
Provincial health statistics				
HIV prevalence^e^	25.1	19.8	4.6	9.4
Infant mortality rate^c^^,f^	63	68	41	82
% women using modern FP method^c^^,f^	18.2	32.8	5	2.9
% fully vaccinated child (12–23 months)^c^^,f^	76.3	87.9	66.3	58.5
% pregnant women with 1+ ANC visits^c^	96.6	99	92.9	96.1
Institutional deliveries^c^	70.7	88.3	53.3	36.2

* Maputo refers to the province, not the city

a Ministry of Health (MISAU) 2012.

b Ministry of Health (MISAU) 2013.

c Ministry of Health (MISAU), National Statistics Institute (INE), ICF International (ICFI). Demographic & Health Survey 2011. Calverton, Maryland, USA: MISAU, INE, ICFI. Calverton, Maryland, USA: MISAU, INE, ICFI.

e The National Statistics Institute (INE), and ICF Macro. 2009–2010. National Survey on Prevalence, Behavioural Risks and Information about HIV and AIDS in Mozambique.

f MISAU, INE, UNFPA *et al.* 2009.

As an observational study, the selection of an appropriate control group is essential. We chose neighbouring provinces to serve as controls and assessed their comparability using a propensity score matching technique on a health facility level (Table 2). Facility-level data for matching controls was obtained using the national facility survey, which is annually conducted by the GRM. This dataset includes descriptive variables such as the number of staff, the number of beds, the availability of water and electricity supply, and the presence of a financial institution in the area. In addition, control and intervention provinces received comparable levels of technical assistance and external funding, with similar systems for data monitoring, except intervention provinces received these resources through a combination of input and output-based financing. Finally, a detailed policy analysis revealed that no uneven confounding changes to policy or clinical protocol (such as Option B+ PMTCT rollout or CD4 thresholds for initiating ART) occurred in any of the four study provinces during the study period (Table 1). This study was approved by the CDC Ethical Review Board in August 2013. The protocol was also approved by the Comite nacional de Bioetica para Saude, Ministerio da Saude Mozambique in October 2013.

### Data

Data for this study were drawn from three principal sources. In the intervention sites (Gaza and Nampula), data for PBF indicators were extracted from the quarterly PBF invoices produced by each facility for a maximum of an 11-quarter study period (January 2011–September 2013, 2.75 years). Three of the 21 indicators (number of male partners of all women tested for HIV, number of HIV tests administered at the health facility and the number of HIV infected patients lost to follow up who come back for ART treatment) did not meet our inclusion criteria of triangulated data from multiple sources in both the intervention and control provinces, and were not included in the study. In the control sites, HIV indicators were collected using an electronic patient database, a standardized HIV/AIDS information system. For all provinces, data for non-HIV indicators, including the two non-incentivized indicators, were obtained using the national health information system. Combining these data sources, an 11 quarter (33 months or 2.75 years) panel dataset was constructed.

### Data integrity

Multiple layers of data quality checks were performed for both intervention and control provinces. First, monthly reports submitted by PBF facilities were scrutinized for reported discrepancies above two standard deviations. If detected, the indicator was recounted and verified. Second, the routine quarterly verification procedure was conducted in which all indicators at all facilities were verified, and carried penalties for misreporting. Finally, the study team conducted a data audit in the intervention provinces. The audit team randomly selected 25% of the PBF health facilities and audited a random list of five to seven indicators in each of them. No systematic discrepancies were detected in either direction; the data were neither systematically under- nor over-reported. Similarly, Ariel, the NGO implementer in the control regions, verified monthly data reports in control provinces by triangulating (cross-referencing) the data from multiple sources. Control data was counter-verified by external annual audits performed by an independent auditor.

### Econometric methods

Our methodological framework attempts to control for selection bias using a two-stage approach. First, a matching algorithm was implemented to construct a matched comparison group for all PBF facilities using propensity scores (Ho *et al.* 2007). Accuracy of matching was assessed by performing balancing tests at 5% statistical significance (Supplementary Annex S1). Second, a multi-period difference-in-differences (MDID) was applied on the matched sample to obtain average treatment effects (additional information in Supplementary Annex S2). Making full use of the panel dataset, the MDID also allowed us to uncover the lifecycle of the treatment effect over time, such as time to attain peak treatment effect by indicator and degradation of treatment effect over time, by indicator. The combination of these two methods to control for selection bias in observational studies is considered a powerful alternative when experimental data is not available (Blundell and Costa Dias 2000).

Our regression design takes the form of a generalized linear mixed model with both fixed and random effects, fitted using the seemingly unrelated regression technique in STATA statistical software. Bootstrapped standard errors were produced and used for inference.

### Temporal effects

We considered the effect of PBF immediate if a statistically significant difference (*P* < 0.1) was observed after three quarters of exposure (9 months), since the first PBF payment was disbursed well into the second quarter of enrolment. We considered statistically significant differences observed after four or more quarters to be delayed effects.

### Analysis of responsiveness

Rather than evaluating impact based on statistical significance alone, we created a responsiveness index that equally weights magnitude of impact and duration of impact. Magnitude scores were constructed by clustering the magnitude of average treatment effect estimates. Duration scores are a function of the number of quarters of statistical significance observed (*P* < 0.1), with consecutive quarters of significance receiving greater weight (Table 3). The composite score for responsiveness had a possible range between 0 and 2. Based on the observed clustering of scores, we developed four categories of responsiveness, as shown in Table 3.

**Table 3.** Calculating the responsiveness index

Magnitude					**Responsiveness**	
% increase over baseline	100%+	50–99%	10–49%	0–9%	Composite score	Responsiveness category
Score	1	0.67	0.33	0	0	Unresponsive
**Duration**					0–0.67	Low responsiveness
Duration of impact (quarters)	3+ consecutive	2 consecutive	3 + (non-consecutive)	2 (non-consecutive)	0.67–1.33	Medium responsiveness
Score	1	0.5	0.3	0.2	1.33–2	High responsiveness

Using this categorization, we performed two levels of analysis in order to investigate the relationship between reimbursement rate (or price) and the responsiveness of PBF indicators. First, we examined the correlation between the price of an indicator and its responsiveness to PBF (as defined above). Second, we examined the relationship between the provider reimbursement rate for each service to the volume of services provided (price elasticity). We looked at price elasticity, a measure of the responsiveness of the quantity demanded of a good or service to a change in its price, in order to examine the relationship between indicator output and a change in that indicator’s price. Our data for price elasticity are limited: 14 indicators underwent 2 price changes and the remaining 4 underwent one price change.

We also tested the relationship between the level of effort required for each indicator and the responsiveness of that indicator. To determine the level of effort required for each indicator, six consultations were held with a group of clinical experts consisting of two clinical advisors, four clinical technical assistance providers and two district medical directors. We also conducted three group discussions (five participants on average) with health care providers (including nurses, physicians and pharmacy technicians). We asked them to categorize the incentivized indicators into three categories of effort: low, medium and high, taking into account the following factors:

- Complexity of treatment;
- Health worker’s degree of control over outcome;
- Intensity of follow-up required;
- Potential volume of work;
- Volume of other work relative to this indicator;
- The performance of the indicator at the baseline.

Final categorization was made based on a secondary validation workshop held with both clinical experts and providers. Using these categorizations we examined the correlation between the level of effort and responsiveness.

## Results

### Sample size

The results of the propensity score matching are given in Table 4.

**Table 4.** Results of the balancing tests after propensity score matching

Covariates	All		North		South	
	Difference	*P*-value	Difference	*P*-value	Difference	*P*-value
Site type	0.0342	0.6805	−0.1415	0.2747	−0.1701	0.2488
Availability of water	−0.0478	0.5588	−0.1649	0.0849	0.0833	0.5384
Availability of electricity	0.0765	0.3816	−0.0054	0.9594	0.1632	0.2511
Availability of a laboratory	−0.2064	0.1089	−0.4624	0.0019	0.0451	0.8508
Availability of a financial institution	0.061	0.0704	0.08	0.0651	0.0313	0.4589
Availability of a training institution	0.0244	0.2598	0.04	0.1995	0	1
# of maternity beds	−0.4667	0.7507	−1.336	0.4264	−1.0382	0.7209
# of medical health workers	0.4362	0.6511	−1.1507	0.3138	0.8715	0.6414

**Sample size (*N*)**			**Intervention**		**Control**	
	**Total**		Gaza	**Nampula**	**Cabo Delgado**	**Maputo**

After matching	134	30	48	39	17
Before matching	147	35	50	43	19

*Note*: Unmatched facilities were excluded from the study sample.

After matching, 134 out the 147 health facilities were matched, with 87 from the North (48 in the intervention region, 39 in the control region) and 47 from the South (30 in the intervention, 17 in the control), which accounts for approximately 35% coverage of total facilities across the 4 study provinces. Table 4 shows that for all except one covariate (availability of a training institution), the *P*-value of the mean difference is > 0.05, indicating that selection bias on observables was mitigated in the new matched sample.

### Impact of PBF

Tables 5 and 6 show the full results of the impact evaluation. Initiation of HIV-infected pregnant women on ART showed strong improvements with PBF in both regions—on average, a health facility with PBF initiated 9.1 (standard error [SE] 1.3, *P* < 0.001) more HIV-infected pregnant women on ART per quarter than a control facility in the North, and 19.4 (SE 3.8, *P* < 0.001) more women per quarter in the South (Tables 5 and 6). This translates into a 251.6% increase over baseline or 764 more women per quarter across all study-sample facilities in the North, and a 194.6% increase over baseline or 970 more women per quarter in all facilities in the South. Similarly, more women completed four antenatal care (ANC) visits in PBF facilities: in the North, on average, a health facility with PBF saw 176.8 (11.6, *P* < 0.001) more women per quarter completing four ANCs than control facilities, translating into 14 851 women per quarter in the province or a 153% increase over baseline. Similarly, a health facility with PBF in the South saw an average of 88.6 (15.1, *P* < 0.001) more women completing four ANCs per quarter than control facilities, translating into 7442 women per quarter in the province or an 82.4% increase over baseline.

**Table 5.** Average treatment effect of PBF in the North

Indicator	Category	Level of effort	Price	Quarterly average effect per facility (standard error)	Magnitude (% change of baseline)	Onset of effect	Responsiveness score
**High responsiveness**							
HIV-infected pregnant women who initiated ART	PMTCT	Low	$10	9.1*** (1.3)	251.6	Delayed	2
HIV-infected women who received a FP consultation and a modern contraceptive method	PMTCT	Low	$5	13.2*** (1.2)	162.6	Delayed	2
Pregnant women who completed 4 ANC visits	MCH	Low	$2	176.8*** (11.6)	153.6	Immediate	2
Children, born to HIV-infected women, who were tested via rapid test for HIV 9–12 months after birth	Paediatric HIV	Low	$4.20	7.4*** (1.2)	101.2	Delayed	2
Post-natal consultations 3–28 days after birth	MCH	Medium	$1.60	185.7*** (19.5)	64.4	Delayed	1.67
HIV-infected pregnant women who received ARV to reduce mother-to-child transmission	PMTCT	Low	$6.25	8.3*** (0.84)	80.9	Immediate	1.67
**Medium responsiveness**							
Children, born to HIV-infected women, who were tested (PCR) for HIV between 4 and 8 weeks age	Paediatric HIV	Medium	$4.90	3.4*** (0.65)	30.9	Delayed	1.33
Women, excluding HIV-infected women, who received a FP consultation and a modern contraceptive method	MCH	Low	$0.10	219.2***(25.9)	45.5	Delayed	1.33
Facility deliveries	MCH	Low	$3	66.0*** (7.4)	24.3	Immediate	1.33
Children who received full vaccination for BCG, DPT, polio and measles in the first 9 months	MCH	Low	$1.80	98.1*** (14.5)	42.2	Delayed	1.33
HIV-infected children 0–14 years of age alive 12 months after initiating ART	Paediatric HIV	High	$11.20	1.21*** (0.35)	34.5	Delayed	1.17
HIV-infected patients who initiated Isoniazid to prevent TB	Adult HIV	Medium	$2	38.7*** (5.9)	89.9	Delayed	1.17
Adults co-infected with HIV and TB who initiated ART treatment for the first time	Adult HIV	High	$2.80	1.9*** (0.36)	60.5	Delayed	1.17
Children with acute malnutrition who completed treatment and satisfied the defined clinical criteria for discharge	MCH	Medium	$1.50	1.7** (0.62)	29.6	Delayed	0.83
**Unresponsive indicators**							
HIV-infected children 2–14 years of age who initiated ART treatment for the first time	Paediatric HIV	High	$7	–	–	–	0
HIV-infected children 0–23 months of age who initiated ART treatment for the first time	Paediatric HIV	High	$7.70	–	–	–	0
HIV-infected adults (>15 years) alive 12 months after initiating ART	Adult HIV/TB	High	$8	–	–	–	0
HIV-infected adults (excluding pregnant women) who initiated ART treatment for the first time	Adult HIV/TB	High	$4	–	–	–	0

*Note*: BCG: Bacille Calmette-Guerin; DPT: Diphtheria, tetanus, and pertussis vaccine; PCR: polymerase chain reaction.

Level of significance: *P ≤ 0.05; **P ≤ 0.01; ***P ≤ 0.001

Prices are reflective of quarter 11.

**Table 6.** Average treatment effect of PBF in the South

Indicator	Category	Level of effort	Price	Quarterly average effect per facility (standard error)	Magnitude (% change of baseline)	Onset of effect	Responsiveness score
**High responsiveness**							
HIV-infected pregnant women who initiated ART	PMTCT	Low	$10	19.4*** (3.8)	194.6	Delayed	2
HIV-infected women who received a FP consultation and a modern contraceptive method	PMTCT	Low	$5	58.8*** (12.4)	221.7	Delayed	2
Pregnant women who completed four ANC visits	MCH	Low	$2	88.6*** (15.1)	82.4	Delayed	1.67
**Medium responsiveness**							
HIV-infected children 0–23 months of age who initiated ART treatment for the first time	Paediatric HIV	High	$7.70	3.2*** (0.6)	45.2	Delayed	1.33
Children, born to HIV-infected women, who were tested via rapid test for HIV 9–12 months after birth	Paediatric HIV	Low	$4.20	12.2*** (3.1)	54.6	Delayed	1.17
Children, born to HIV-infected women, who were tested (PCR) for HIV between 4 and 8 weeks age	Paediatric HIV	Medium	$4.90	17.2*** (2.2)	53.3	Delayed	1.17
Children with acute malnutrition who completed treatment and satisfied the defined clinical criteria for discharge	MCH	Medium	$1.50	4.0** (1.4)	64.6	Delayed	0.87
HIV-infected children 0–14 years of age alive 12 months after initiating ART	Paediatric HIV	High	$11.20	4.2***(0.94)	34.5	Delayed	0.83
**Low responsiveness**							
Post-natal consultations 3–28 days after birth	MCH	Medium	$1.60	40.7* (17.2)	24.8	Delayed	0.53
**Unresponsive Indicators**							
Women, excluding HIV-infected women, who received a FP consultation and a modern contraceptive method	MCH	Low	$0.10	–	–	–	0
HIV-infected pregnant women who received ARV to reduce mother-to-child transmission	PMTCT	Low	$6.25	–	–	–	0
HIV-infected patients who initiated Isoniazid to prevent TB	Adult HIV/TB	Medium	$2	–	–	–	0
HIV-infected children 2–14 years of age who initiated ART treatment for the first time	Paediatric HIV	High	$7	–	–	–	0
Facility deliveries	MCH	Low	$3	–	–	–	0
Children who received full vaccination for BCG, DPT, polio and measles in the first 9 months	MCH	Low	$1.80	–	–	–	0
HIV-infected adults (>15 years) alive 12 months after initiating ART	Adult HIV/TB	High	$8	–	–	–	0
HIV-infected adults (excluding pregnant women) who initiated ART treatment for the first time	Adult HIV/TB	High	$4	–	–	–	0
Adults co-infected with HIV and TB who initiated ART treatment for the first time	Adult HIV/TB	High	$2.80	–	–	–	0

*Note:* BCG: Bacille Calmette-Guerin; DPT: Diphtheria, tetanus, and pertussis vaccine; PCR: polymerase chain reaction.

Level of significance: *P ≤ 0.05; **P  ≤ 0.01; ***P  ≤ 0.001

Prices are reflective of quarter 11

PBF had no spill-over effect on the two analysed non-incentivized services. There was no significant impact on the number women who completed malaria prophylaxis in the intervention areas, while well-child visits for children under the age of four demonstrated increasing tendencies in the North and remained unchanged in the South (data not shown).

### Responsiveness

Of the 18 incentivized PBF indicators, 14 in the North (77%) and 9 in the South (50%) were responsive to PBF (Tables 5 and 6). In both regions, at least half of the indicators demonstrated an increase in average output of > 50% relative to baseline. That said, PBF had a stronger magnitude of impact in the North.

The most responsive indicators pertain mainly to HIV-infected women, their children and MCH services. In both regions, the three indicators that were among the most responsive to PBF included: HIV-infected pregnant women initiating ART, family planning (FP) consultations for HIV-infected women and number of ANC visits for women overall.

Adult HIV indicators ranked at the lower end of the responsiveness scale in both regions. Four ART-related indicators were not responsive at all to PBF, including the critical adult initiation and treatment continuation (sustained access to ART) indicators. Interestingly, while the initiation of ART treatment among pregnant women experienced strong improvements, the initiation of ART treatment among both paediatric patients and adults did not respond to PBF.

There was heterogeneity between regions in the responsiveness of the remaining service indicators: six service indicators responded to PBF in one region, but not in the other. Number of new children age 0–23 months initiating ART did not improve in the North, while it achieved medium responsiveness in the South. The remaining five indicators (number of women, excluding HIV-infected women, who received an FP consultation, number of HIV+ pregnant women who received ARV (PMTCT), number of adults co-infected with TB who initiate ART, number of children completely vaccinated at 9 months, and number of facility deliveries) experienced the opposite: they did not improve in the South, but had medium to high responsiveness to PBF in the North.

There was no statistically significant relationship between the price and responsiveness of PBF indicators: the correlation coefficient for the North is −0.22 (*P* = 0.39), and 0.25 (*P* = 0.31) for the South. In addition, the price elasticity analysis showed that there was no statistically significant correlation between changes in indicator price and the level of output for any of the 18 indicators.

The results showed a statistically significant relationship between level of effort and responsiveness in the North with a spearman’s rank correlation coefficient of −0.67 (*P* = 0.002), and a non-significant relationship in the South (−0.34, *P* = 0.17). The relationship between responsiveness and the level of effort to produce each indicator was inversely correlated in both regions.

### Temporal effects

Overall, the results show that the onset of sustained PBF impact does not occur immediately (Tables 5 and 6). In the North, it took an average of 14 months of exposure for PBF to take effect, compared with 20 months in the South. Only three indicators responded immediately to PBF in the North, while only one indicator responded immediately in the South. MCH and PMTCT indicators tended to respond the fastest to PBF.

**Figure 1.**
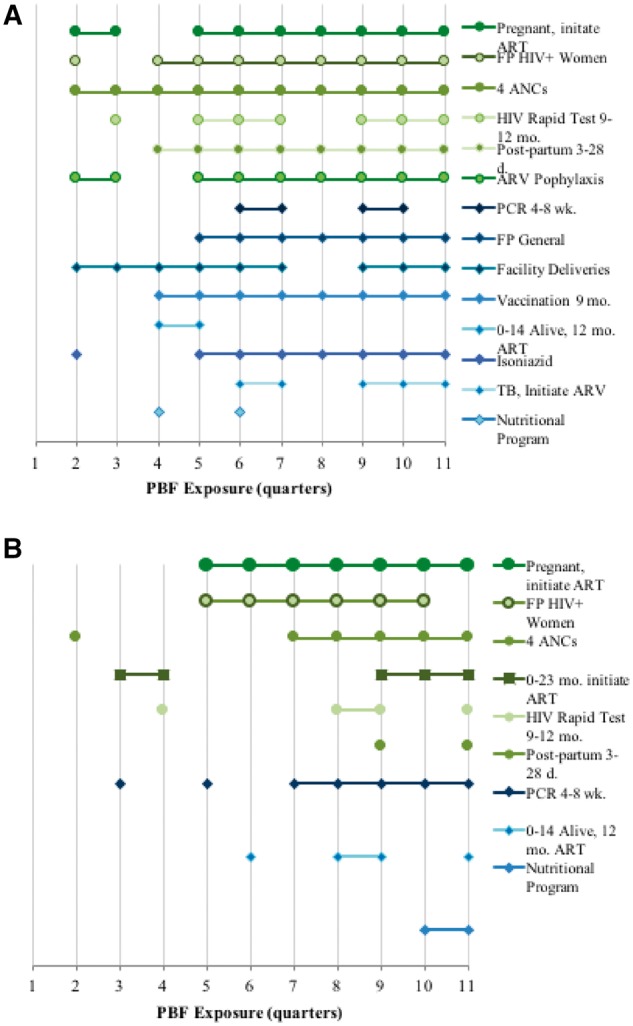
Duration of PBF effect in the North (a) and the South (b)

The onset of PBF impact on the two most responsive indicators (number of HIV-infected pregnant women who initiate ART and the number of FP consultations for HIV infected women) is consistent across the two regions. It took five quarters (15 months) for PBF to elicit a statistically significant (*P* < 0.1) positive response in the number of pregnant women who initiate ART in both regions. Similarly, it took four quarters (12 months) for FP consultations for HIV-infected women to improve in the North, and five quarters in the South. The effect of PBF tended to persist over time, especially in the North. Once PBF took effect in the North, the effects were sustained for 11 out of the 14 indicators (Figure 1). In the South, half of the indicators demonstrated a sustained impact after initial onset.

The three most responsive indicators (FP for HIV-infected women, women receiving four ANC visits and initiation of ART for HIV-infected pregnant women) responded faster in the North than the South, but in all cases demonstrated a sustained impact once a statistically significant positive impact was observed.

## Discussion

The results of this study show that, in the context of this program, PBF can result in sustained and large increases in health service outputs. We found this to be the case particularly for MCH, PMTCT and paediatric HIV services, which were much more responsive than adult HIV/TB services. Evidence from other PBF evaluations supports its positive impact on MCH and PMTCT (Basinga *et al.* 2011; Bonfrer *et al.* 2014; Binagwaho *et al.* 2014; Zeng *et al.* 2014; de Walque *et al.* 2015; Skiles *et al.* 2015; Van de Poel *et al.* 2015).

Our study is the first impact evaluation to rigorously assess how impact changes as a function of exposure time. We found that, on average, PBF takes six quarters before impact can be observed. However, once impact is observed, we found that it is generally sustained. In Mozambique, the lag between program initiation and results could be due to the time required for health system actors to understand and internalize how PBF works, develop strategies to improve indicators, and organize systems and processes to achieve results. Once this occurs, it is plausible that a new equilibrium is set, such that increased drive and efficiency become the norm. Thus, countries interested in pursuing PBF should recognize that achieving the benefits of PBF will take time. Along those same lines, impact evaluations of PBF programs should assess at least six quarters of implementation.

The magnitude of impact shown in this study demonstrates strong improvements across many indicators (Tables 5 and 6). We reviewed eight rigorous PBF impact evaluations conducted over the last decade (Rwanda, Cameroon, Tanzania, Cambodia, DRC and Burundi) and found increases in the quantity of services provided of between 4 and 132% whereas the magnitude of increases found in this study were between 16 and 252% (Basinga *et al.* 2011, Huillery and Seban 2013; Bonfrer *et al.* 2014; Falisse *et al.* 2014; Matsuoka *et al.* 2014; Binyaruka *et al.* 2015; Rudasingwa *et al.* 2015; O Zang *et al.* unpublished). Improvements were pronounced for MCH, as well as some indicators pertaining to HIV. This suggests that PBF is a particularly effective strategy for improving health outcomes in these service areas.

Interestingly, our study found that PBF indicators were not sensitive to the prices set within the PBF model. Providers did not prioritize more expensive indicators over less expensive ones, nor did they respond to changes in individual indicator prices. At the same time our study found that indicators that required less level of effort appeared more responsive than those that required more. This suggests that health workers do not prioritize indicators on price alone—they take into account the total cost of effort, associated opportunity costs and likelihood of success. Thus, it is essential for PBF programs to rigorously assess the determinants of responsiveness for each indicator to develop more effective pricing schedules.

The results of this study demonstrate that the context in which PBF is applied is a strong determinant of its success. Even within the same country, a third of the analysed indicators showed differences in responsiveness when comparing the Northern and the Southern region. Moreover, the magnitude of improvements varied greatly across provinces. Many contextual factors could have contributed to uneven performance. For instance, baseline rates for complete vaccination and facility deliveries were substantially higher in the South compared with the North (Ministry of Health (MISAU) 2011). It is therefore not surprising that these two indicators performed well in the North but not the South, as the margin for improvement in the South was smaller. Similarly, Gaza is among the highest HIV prevalence provinces in the country (25.1%), compared with 4.6% in Nampula (National Institute of Health 2009). Accordingly, the country’s ambitious Acceleration Plan to expand ART access and availability, which coincided with the implementation of PBF, could have dampened the impact observed in Gaza. Therefore, we argue that simply picking indicators because they represent a national or international priority, or that they have been successfully applied in other contexts, is a suboptimal strategy to designing a PBF program. Instead, we argue that it is essential for countries to robustly assess the political, cultural, epidemiological and market context in which PBF is to be applied, and to select indicators that will respond best within that complex context.

This study has several limitations. Our evaluation framework employed both propensity score matching and difference-in-differences to control for confounders arising from the differences between intervention and control provinces. However, structural characteristics (such as number of pregnant women in the province) could be rate-limiting factors that influence output. In addition, intervention provinces received both input and output financing and, thus, our results cannot discern whether the impact observed is from PBF alone or the complementarity of PBF with input financing. Finally, we have no way of determining the extent to which improvements in the intervention group are related to better reporting versus better performance.

Our study highlights the critical need for further research into the broader effects of PBF. Qualitative research is needed to understand the contextual factors that led to differences in responsiveness across regions and level of effort, why and how PBF led to improvements, and how the broader health system (governance, human resources, pharmaceutical management, quality of care) has adjusted to PBF. Finally, while the study shows that PBF is an effective complement to input financing, the cost-effectiveness of the overall approach requires rigorous evaluation.

## Conclusion

In the context of this study, our results show PBF is an effective strategy for driving down the HIV epidemic and advancing primary care service delivery than input financing alone. Our study did not find any negative effects of PBF vis-à-vis pure input financing, nor did we observe spill-over effects (positive or negative) in the examined non-PBF indicators. Thus, large-scale external financing programs in Mozambique should consider adopting an output-based model, tailored to each provincial context. That being said, the fact that some indicators were not responsive shows that PBF is not a silver bullet. Rather, this study highlights the essential need for policymakers to carefully examine their own contexts to determine whether PBF could be an effective solution relative to other health system and financing mechanisms.

## Supplementary Data

Supplementary data are available at *HEAPOL* online.

## Disclaimer

The findings and conclusions in this report are those of the author(s) and do not necessarily represent the official position of the Centers for Disease Control and Prevention or the Elizabeth Glaser Pediatric AIDS Foundation.

## Funding

This study was supported by the Presidents’ Emergency Program for AIDS Relief (PEFPAR) through the Centers for Disease Control and Prevention (CDC), Project Fortalecer Cooperative Agreement U2GGH000422-01. CDC provided grant funding to the Elizabeth Glazer Paediatric AIDS Foundation (EGPAF) to implement the PBF program. EGPAF, who granted funding for this study, had no role in the study design, data analysis, data interpretation or writing of the report. The CDC author contributed to the analysis and interpretation of results, but had no role in the design of the study, the selection of settings considered, strategies evaluated or the development of the econometric models. The corresponding author had full access to all the data in the study and had final responsibility for the decision to submit for publication.

*Conflict of interest statement*. Yogesh Rajkotia reports grants from the Elizabeth Glazer Paediatric AIDS Foundation, from null, during the conduct of the study and Jobarteh reports previous employment at the Centers for Disease Control. All other authors declared that they have no conflict of interest. 

## Supplementary Material

Supplementary Data
