# Supplementary material for: The effect of a performance-based financing program on HIV and maternal/child health services in Mozambique—an impact evaluation
Source: Health Policy Plan. 2017 Oct 23;32(10):1386–96. doi: 10.1093/heapol/czx106 (PMC5886140; doi:10.1093/heapol/czx106)
Supplement: Supplementary Data [file supplementary_file_czx106.docx]

**Annex 1. Sensitivity Analysis**

**Robustness of Match**

To ensure that the match between the control and intervention group did not change over time, balancing tests are performed on covariates measured in the last quarter (the 11th quarter) using the same matching variables used at baseline. The results of the balancing tests show the distribution of the covariates across the intervention and the control groups are as similar as they were in 2011 (the mean-differences are statistically equal to zero with P-values showing similar results as in 2011). This implies that the value of comparison groups did not deteriorate during the subsequent 11-quarter periods of the study. Moreover, regressions using these new measurements of covariates did not change the main results. This confirms that our results are robust to matching and are insensitive to any possible temporal effect on the covariates during 11 quarters of the program.

**Biases Due to Baseline**

Data prior to the initiation of the PBF program is not available for all sites, thus the first quarter of exposure for a given health facility serves as baseline. To test for any bias this may introduce, we repeat the econometric analysis using the second, third, and fourth quarters as baseline and examine how the magnitude and direction of our estimates respond. This analysis revealed no discernable bias in either direction.

**Annex 2: Econometric Methods**

**Overall Econometric Framework**

Our methodological framework attempts to control for selection bias using a two-stage approach. First, a matching algorithm is implemented to construct a matched comparison group for the treated using propensity scores. The purpose of matching is to adjust the data prior to parametric analysis such that the relationship between the treatment status (being selected to receive PBF) and the factors that explain the outcome is eliminated or sufficiently reduced. Second, average treatment effects are estimated by applying a difference-in-differences (DiD) model on the matched sample. The purpose of the DiD model is to eliminate the effects of common temporal confounders (such as national policy changes), as well as account for permanent differences between the control and treatment groups.

Our approach goes beyond the traditional method of simply estimating average treatment effects over the study period. Instead, we make full use of our panel dataset and structure our model as a multi-period DiD, allowing us to uncover the lifecycle of the treatment effect over time in a nonlinear way. This allows us to examine temporal effects such as time to attend peak treatment effect by indicator and degradation of treatment effect over time by indicator.

**Propensity Score Matching**
Similar to randomization in a classical experiment, matching aims to balance the distributions of all relevant, pre-treatment characteristics in the treatment and comparison group in order to achieve independence between the outcomes and the assignment to treatment. We therefore need to assess the accuracy of the matching by performing balancing tests. We perform these tests by computing the mean differences of the covariates and assessing their significance using their p-values. A p-value lower than 0.05 provides evidence that the sample moments of two variables are not equal at a 5% statistical significance.

After choosing the final matched sample with maximum balance, reduced heterogeneity, and a large number of observations included, the parametric analysis can be performed. The matched data helps obtain accurate causal effect estimates, such as with small bias and variance, less sensitive to parametric functional form assumptions for the outcome equation (Ho et al. 2007). Next, we present and discuss the estimation procedure for the outcomes equation.

**Multi-period Difference-In-Difference Estimator**

The structural model we use for this analysis is a multi-period difference-in-difference (MDiD) estimator (Imbens and Wooldrige 2009; Imbens and Wooldrige 2010, Jones 2007). The MDiD-estimator extends the classic two-period DiD model by estimating average treatment effect between the baseline period (one quarter of exposure) and subsequent quarters of exposure to PBF (Heckman et al. 1998). Practically, this is accomplished by adding dummy variables to account for the number of quarters of exposure to PBF. The model is also specified with a set of control variables obtained from the national facility survey.

Since there are multiple outcomes of interest (18 PBF and 2 non-PBF indicators), most of which have been produced at the same locations and during the same time periods, there are possibly unobserved interactions between them that cannot be accounted for by separate regressions. To account for possible spillovers, we use the seemingly unrelated regression (SUR) technique to fit the regression models on all outcomes at the same time (Greene 2012). This way, cross-output correlations can be included and a more efficient variance-covariance matrix for the treatment effects can be obtained. This allows us to examine the lifecycle of the treatment effect over time, e.g. by evaluating temporal effects such as time to attend peak treatment effect by indicator, degradation of treatment effect over time by indicator, etc.

Lastly, to assure the accuracy of our treatment effect estimates, in terms of significance, bootstrapped standard errors are performed and used for inference. The model is estimated using Stata software that has procedures that allow SUR estimation of the effects and calculate appropriate tests and associated bootstrapped P-values to establish statistical significance.

**References:**

Blundell R, Costa Dias M. Evaluation methods for non‐experimental data. *Fiscal Studies* 2000;**21**(4):427-68

*Econometrica* 1998;**66**:1017-98

Greene W. *Econometric Analysis.* 7^th^ Edition. Boston: Prentice Hall, 2012

Heckman J, Ichimura H, Smith J et al. Characterizing Selection Bias Using Experimental Data.

Ho DE, Imai K, King G et al. Matching as nonparametric preprocessing for reducing model dependence in parametric causal inference. *Political Analysis* 2007;**15**(3):199-236

Imbens G, Wooldrige J. Recent Developments in the Econometrics of Program Evaluation. J*ournal of Econometric Literature* 2009;**47**(1):5-86

Imbens G, Wooldrige J. What’s New in Econometrics? Lecture Notes 10. NBER. Summer 2010

Jones, A.M. *Applied econometrics for health economists: a practical guide*. Radcliffe publishing. 2007.

Rosenbaum P, Rubin D. The Central Role of the Propensity Score in Observational Studies for Causal Effects. *Biometrika* 1983;**70**:41-50
